# Artificial Womb Technology for Extremely Premature Neonates: Preclinical Neurodevelopmental Outcomes

**DOI:** 10.3390/children13010047

**Published:** 2025-12-30

**Authors:** Felix R. De Bie, Chelsea J. Zhang, Sharla M. Rent, Jeffrey B. Russ, Kevin Ig-Izevbekhai, Ryan M. Antiel

**Affiliations:** 1 Department of Surgery, Division of Pediatric Surgery, Duke University, Durham, NC 27705, USA; 2 Department of Pediatrics, Division of Neonatology, Duke University, Durham, NC 27705, USA; 3 Department of Pediatrics, Division of Pediatric Neurology, Duke University, Durham, NC 27705, USA

**Keywords:** extreme prematurity, artificial womb technology, neurodevelopment

## Abstract

In this narrative review, we examine current neurological and neurodevelopmental outcomes associated with extreme prematurity, specifically in infants born between 22 and 24 weeks of gestational age. In addition, we explore the scientific and clinical rationale for the development of artificial womb technology and provide an overview of the current state of the art in preclinical models. For each model, we review and analyze the neurological outcomes, including structural, developmental, and functional outcomes, evaluating their limitations in anticipation of imminent clinical translation.

## 1. Introduction

### 1.1. Extreme Prematurity

The World Health Organization (WHO) reports that approximately 13.4 million infants worldwide (nearly one in ten) are born premature at less than 37 weeks of gestation [1]. Within this population of premature infants, 4.2% are born extremely premature at less than 28 weeks of gestation [2,3]. Notably, there has been little or no change in both the global and regional preterm birth rates in the last decade [3]. Despite significant advancements in neonatal critical care, extreme prematurity, especially near the limit of viability, remains a leading cause of infant morbidity and mortality [4].

The limit of viability is the earliest estimated gestational age (EGA) at which an infant has a chance of survival outside of the maternal womb if provided with intensive medical care [5]. The limit of viability correlates with multiple factors including EGA, sex, birth weight, singleton vs. twin pregnancy, antenatal corticosteroid exposure, available healthcare resources, and ultimately the willingness of the medical team to offer resuscitation [6,7]. The World Health Organization (WHO) has considered 22 weeks EGA as the lower limit of viability for statistical purposes [8].

### 1.2. Survival

Technological advancements and increased use of neonatal intensive care (NICU) have significantly improved the survival of extremely premature infants (EPI) born at the limit of viability in the United States. In 2022, 73.7% of infants born at 22 weeks EGA underwent active resuscitation, with a survival rate of 36.1% at hospital discharge [9]. While this is markedly improved from 10.9% survival at discharge between 2013 and 2018, disability-free survival remains just 6.2% for EPIs born at 22 weeks EGA [9,10]. At 25 weeks EGA, 99.8% of infants underwent resuscitation, with a survival rate of 82.0% at discharge, and 40.9% achieved disability-free survival [9].

Internationally, greater active perinatal management has resulted in improved outcomes for EPIs. In Sweden, higher perinatal activity was accompanied by an increase in survival at 22 weeks’ gestation from 10% (2004–2007) to 39% (2014–2019) [11]. The German Neonatal Network saw similar results, with 22–24 weeks’ gestation survival rates increasing from 53% (2011–2013) to 64% (2014–2016) [12]. In Japan, national data from 2018 to 2020 showed active resuscitation for 85% of infants born at 22 weeks and 98% at 23 weeks, with corresponding survival rates among those resuscitated of 63% and 80%, respectively [13].

### 1.3. Pulmonary Morbidity

The primary morbidity among EPIs is respiratory failure due to structural pulmonary immaturity causing bronchopulmonary dysplasia (BPD) [14]. Toward the end of the canalicular stage, around 24 weeks’ gestation, lung development transitions into the saccular stage, characterized by the development of primitive alveolar sacs [15]. EPIs born before 24 weeks have very limited alveolar surface area and immature surfactant production, hampering effective oxygenation and ventilation [16]. As a result, EPIs require mechanical ventilation for adequate gas exchange [17,18]. However, the immature lung is highly susceptible to injury from barotrauma, volutrauma, and oxygen toxicity, which can trigger inflammation and disrupt normal lung growth [19,20]. While interventions such as antenatal corticosteroids, early surfactant therapy, or combination therapy of surfactant with intratracheal corticosteroids can reduce mortality and acute respiratory morbidity, they do not reduce rates of BPD amongst EPIs, reflecting the profound vulnerability of the fetal lung [21,22,23,24]. Indeed, a meta-analysis demonstrated that over the last 30 years, the prevalence of BPD in EPIs has not decreased, despite improved NICU care [25].

### 1.4. Gastro-Intestinal Morbidity

Gastro-intestinal tract development is also impacted by extremely preterm birth and very low birth weight (VLBW), with 7.6% of VLBW neonates affected by necrotizing enterocolitis (NEC), of which 41.8% require surgical intervention [26]. While the pathophysiology remains incompletely understood, immature gut development and immune system function, combined with insufficient blood flow, are likely contributors [27].

### 1.5. Retinopathy of Prematurity

Another condition affecting EPIs is retinopathy of prematurity (ROP), a vasoproliferative retinal disorder and the primary cause of childhood blindness. Exposure to higher oxygen levels in the NICU (to support blood oxygenation) can suppress normal vascular growth in the weeks immediately following birth [28]. Despite early treatment with cryotherapy, laser photocoagulation, and anti-vascular endothelial growth factor, an estimated 32,300 infants per year globally suffer from impaired vision due to ROP [29,30].

### 1.6. Neurologic Sequelae

Major neurological complications in EPIs include intraventricular hemorrhage (IVH) and post-hemorrhagic hydrocephalus, preterm brain injury including periventricular leukomalacia (PVL), and acute symptomatic seizures. Longer-term sequelae of these injuries include developmental delay, cerebral palsy (CP), and epilepsy [31,32]. Chronic hypoxemia due to BPD further contributes to smaller total brain and cerebral white matter volumes, as well as lower brain growth velocity from term-equivalent age to 18 months corrected age [33]. Comorbid inflammatory conditions, such as exposure to chorioamnionitis, necrotizing enterocolitis, or sepsis, exacerbate the risk of preterm brain injury and likely worsen developmental outcomes [34].

IVH is a potentially life-threatening bleed into the germinal matrix secondary to immature and structurally fragile vessels and impaired autoregulation of cerebral blood flow in EPIs, classified into four grades based on the quantity and impact of the bleeding [35,36]. Grade I and II are mild with bleeding occupying less than half the ventricle, while more severe grade II bleeds, grade III bleeds, and grade IV bleeds increase the risk of post-hemorrhagic hydrocephalus and the need for cerebrospinal fluid diversion. Additionally, grade IV bleeds expand into the parenchyma itself, leading to direct parenchymal injury [37]. IVH affects 28.8% of premature infants born before 30 weeks EGA, often occurring within the first 24 h of life [38]. Depending on the grade and associated brain injury, IVH can result in lifelong developmental delay, cognitive impairment, cerebral palsy, and hearing and visual impairments [35,39]. IVH risk in EPIs is multifactorial, with perinatal infection, hemodynamic instability, and respiratory distress requiring mechanical ventilation [36]. A systematic review and meta-analysis concluded that prophylactic indomethacin and antenatal corticosteroid administration are associated with a reduced risk of severe IVH [40]. Volume-targeted ventilation, early use of erythropoiesis-stimulating agents, and prophylactic ethamsylate are also protective in preterm infants, albeit with low certainty of evidence [40,41].

Preterm brain injury disproportionately affects the white matter, particularly the periventricular white matter, commonly resulting in PVL. Preterm white matter injury is multifactorial; however, it is thought to be driven predominantly by the selective susceptibility of white matter oligodendrocyte precursor cells to hypoxia–ischemia. PVL primarily affects the periventricular end-arterial watershed zones vulnerable to hypoxia–ischemia near the occipital and temporal horns and periventricular fasciculi. White matter injury and PVL can affect up to 75% of EPIs compared to 5.7–11.6% of term infants [42]. White matter injury can result in long-term sequelae including cognitive, behavioral, visual, and motor deficits. In fact, PVL is the primary cause of cerebral palsy in preterm infants [42].

### 1.7. Neurodevelopmental Outcomes

There is wide variability in neurodevelopmental disability among EPIs including cerebral palsy and motor delay, visual and hearing impairments, intellectual disabilities, learning disorders, psychiatric conditions, and attention-deficit and autism spectrum disorders [43]. The pathogenesis is complex, stemming from both cerebral underdevelopment and superimposed acute acquired brain injuries [44].

The prevalence of cerebral palsy (CP) is high, 48.4% at 22 weeks and 11.9% at 26 weeks’ gestation, and presents with varying degrees of muscle spasticity, involuntary movements (dyskinesia), and lack of coordination and balance (ataxia) [10,45]. Sixty-one percent of EPI patients with CP have cognitive impairment [45]. Furthermore, at two years follow up, moderate-to-severe CP was seen in 8.4% of EPIs [10]. Additionally, by age two, 33% of survivors born at 22 weeks EGA, 25% of those born at 23 weeks EGA, and 10% of those born at 26 weeks EGA suffered significant cognitive delays, deafness, or blindness [46]. CP outcomes vary across countries and care settings, thus reflecting variability in the management of EPI internationally [47].

Attention-deficit hyperactivity disorder (ADHD), a disorder estimated to affect 5% of children worldwide, has increased prevalence among EPIs [48]. A longitudinal Swedish cohort study reports a prevalence rate of 12.1% for ADHD among EPIs born between 22 and 27 weeks EGA, compared to 4.5% among infants born at term [49]. In the same study, autism spectrum disorder (ASD) was also more prevalent in EPIs at around 6.1%, compared to 1.4% of infants born at term [50]. Extreme prematurity was also found to be a risk factor for the development of psychiatric and mental health disorders, with anxiety being the most common, at 14.0% of all infants born before 30 weeks EGA compared to 4.0% of those born at 37 weeks or greater [51].

Studies evaluating academic attainment in high school demonstrated lower cognitive ability, reduced reading and mathematics proficiency, and increased requirements for additional academic support in EPIs [52,53]. While some observational studies have demonstrated less visual and hearing impairments over time, the prevalence of cognitive impairment increases with increasing survival of EPIs [54].

Considering the high mortality and substantial morbidity of being born extremely preterm, reimagining how we treat EPIs is necessary. Artificial womb technology (AWT) is an emerging technology proposing to maintain EPIs in a fetal physiologic state to allow for continued maturation by mimicking a uterine, womb-like environment. We explore the rationale and historical development of AWT before reviewing the different preclinical AWT models, focusing on their neurologic outcomes.

## 2. Artificial Womb Technology

### 2.1. Rationale for Artificial Womb Technology

Gas ventilation in EPIs disrupts normal lung development [55]. Between 22 and 24 weeks of gestation, at the threshold of viability, the lungs are still in the late canalicular stage, a period marked by an underdeveloped alveolar network and a thick barrier between alveoli and capillaries [16]. As argued above, the immature anatomy and current therapies result in inefficient gas exchange and ultimately lead to respiratory failure [56]. The objective behind artificial placenta (AP) and artificial womb technologies (AWTs) is to bypass the need for immediate lung-based gas exchange, allowing for continued organ development, particularly the lungs, outside the womb. This novel therapeutic framework is proposed as a way to improve survival and reduce complications in this fragile population.

### 2.2. Model Development

The concept of supporting EPIs through extracorporeal oxygenation first emerged in the early 1950s, coinciding with the advent of rudimentary oxygenator systems [57]. In a landmark experiment in 1958, Westin and colleagues connected the umbilical vessels of seven previable human fetuses to a film oxygenator within a temperature-controlled perfusion chamber, extending their survival for as long as 12 h [58]. Following this, researchers transitioned to animal models to refine the technique, exploring a range of oxygenators, pumps, fluid environments, vascular access strategies, and circuit designs. These iterative modifications progressively enhanced oxygenation efficiency and prolonged survival [59]. The 1970s saw major progress in neonatal medicine, including maternal antenatal corticosteroids [60], exogenous surfactant therapy [61,62,63], and the use of positive-pressure ventilation [64,65], which led to a temporary decline in AWT research. However, by the 1990s, the growing awareness of the shortcomings of conventional treatments reignited interest in AWT development, with a renewed focus on technologies that more closely replicate fetal and placental physiology. Key advances such as eliminating pumps from circuits, engineering low-resistance oxygenators, achieving stable umbilical vessel cannulation, and maintaining the fetus in sterile fluid immersion have brought AWT systems closer to mimicking the natural intrauterine environment. These innovations have steadily improved survival rates and reduced complications during extracorporeal support [59].

## 3. Current Preclinical Models of AWT

Multiple research teams have designed varying models of AP and AWT systems. While the terms are often used interchangeably, they actually refer to distinct approaches that differ in how closely they replicate the physiological conditions of the fetus and placenta. AWT systems, in particular, go beyond replacing the placenta’s essential roles of oxygenation and nutrient delivery by submersing the fetus in a temperature-controlled fluid environment and relying exclusively on umbilical vessel access. In the following sections of this narrative review, we examine five recently developed models, all of which have had their results published in the last five years, and we review their general performance and the reported neurologic outcomes. One caveat is the fact that most AWT devices have been studied in fetal lambs, whose germinal matrix matures earlier than in humans, presumably rendering lambs less vulnerable to IVH at equivalent gestational stages [66]. A summary of the key features of each system is provided in Table 1.

### 3.1. The Children’s Hospital of Philadelphia (USA)

#### 3.1.1. Model

The EXTra-uterine Environment for Neonatal Development, or EXTEND, system was developed in a fetal lamb model. It features a pumpless arterio-venous circuit with a low-resistance oxygenator, directly connected to the umbilical vessels. The fetonate is insulated within a sterile fluid-filled environment, fully immersed in synthetic amniotic fluid [86]. The primary advantage of the EXTEND platform lies in its physiological fidelity: the pumpless arterio-venous setup, exclusive umbilical cannulation, low-resistance oxygenator, and biobag environment closely emulate the natural fetal and placental conditions.

#### 3.1.2. Outcomes

In their landmark 2017 study, Flake and colleagues reported up to 28 days of survival on this circuit in lambs at 106–117 days EGA, followed by successful transition to mechanical ventilation [86]. While on the system, lambs maintained stable hemodynamics without the need for vasopressors, corticosteroids, or external flow regulation, and oxygenation as well as circuit flow remained within physiologic ranges. Due to the sterile conditions and continuous fluid exchange, there were no cases of bacteremia, even in the absence of prophylactic antibiotics.

The lambs demonstrated somatic growth and normal organ development while on circuit. Importantly, their lungs progressed naturally from the canalicular to saccular stage without requiring corticosteroids or tracheal occlusion. When ventilated post circuit, pulmonary function was on par with age-matched control animals [59]. Although a temporary decline in cardiac contractility occurred during the initial week of adaptation to the system, normal cardiac function resumed and was sustained throughout the remainder of support [95]. Mitochondrial assessments confirmed normal metabolic function in vital organs including the liver, kidneys, skeletal muscle, and heart, suggesting preserved fetal metabolic health [94]. Whereas the lambs in the initial studies were noticeably larger (1.0–2.0 kg) than the typical human EPI (<1.0 kg), in a recent study, lambs cannulated at 90–95 day EGA (0.7–1.3 kg) were supported for up to 21 days with adequate oxygenation and normal organ growth [98]. In their latest study, the group reported successful support on the circuit for more than 14 days, with an experimental oxygenator with a small surface area and low priming volume avoiding the need for heparin [105].

#### 3.1.3. Neurologic Outcomes

Brain weight proportionate to total body weight and cerebral parenchymal volume (on MRI) were not different between EXTEND animals compared to in utero controls, suggesting adequate overall brain growth on the circuit [86,96]. MRI analysis showed no difference in myelin deposition levels between EXTEND-supported and control lambs, confirming gradual increasing levels throughout gestation [96].

Gross histological brain analysis did not demonstrate evidence of structural injury, hemorrhage, or infarct, nor did it reveal structural developmental abnormalities when compared to gestational age-matched controls [86,95,96]. Despite the increased maturity of the ovine germinal matrix compared to humans, the absence of IVH in all sheep is reassuring, especially in the context of systemic anticoagulation with heparin while on the circuit.

Microscopically, no differences in myelin density were seen in the internal and external capsules, subcortical white matter, or cerebellum of the brains assessed [86]. Histologic analysis revealed no signs of ischemia, demyelination, or hemorrhage [96]. Neuroinflammation was evaluated via Iba-1, a marker of microglia density in the presence of neurologic injury, and was normal in the cortex, cerebellum and corona radiata of lambs on EXTEND [96]. Assessment of cerebral arteriolar diameter, a downstream marker of cerebral hypoperfusion, did not show significant differences between control and EXTEND lambs in the frontal and parietal cortices [96]. The negative correlation between the mean arteriolar luminal area and mean fetal oxygen delivery over the cannulation period was interpreted as appropriate oxygen-responsive cerebrovascular tone [96].

Cerebral gene expression profiling was performed, comparing preterm lambs supported on EXTEND for three weeks (EGA 125–128 d) to control preterm lambs (EGA 106–107) and control late-preterm lambs (EGA 127 d). Lambs on EXTEND showed greater transcriptomic similarity with the age-matched late-preterm control lambs, thus suggesting continued brain development on the device.

Ultrasonographic assessment of the middle cerebral artery resistance index at 130 days EGA on EXTEND matched reference control ovine values, gradually decreasing with gestation, which further supports adequate cerebral autoregulation on EXTEND [96].

Functional neurologic maturation on EXTEND was observed in the consolidation of sleep–wake cycles while on the circuit [95]. Neurodevelopmentally, animals which transitioned off the EXTEND device did not show gross neurologic deficits, were playful, seeking food, and appropriately responding to vocal and visual stimuli [96].

### 3.2. Tohoku University, Sendai (Japan) and the University of Western Australia, Perth (Australia)

#### 3.2.1. Model

The latest iteration of the Ex-Vivo uterine Environment or EVE system closely mirrors the EXTEND platform in design. It features a pumpless arterio-venous circuit connected via umbilical cannulation, a low-resistance oxygenator, and a sealed, sterile fluid-filled chamber in which the fetus is completely submerged [85]. Specific details of EVE and other systems can be found in Table 1. One of the notable strengths of the EVE system is its ability to support lambs whose size approximates that of a 22–25-week human fetus.

#### 3.2.2. Outcomes

In their most recent study, Usuda, Kemp, and colleagues reported up to 14 days of successful extracorporeal support in fetal lambs cannulated at 95 days’ gestation, with an average body weight of 656 ± 42 g [85]. While the lambs maintained stable systemic circulation within physiological ranges and showed no signs of infection, comparisons with gestational age-matched controls revealed lower organ weight of the lungs, liver, and kidneys, as well as shorter humerus lengths, suggesting impaired somatic growth [85]. Several factors may underlie this finding, including the absence of placental and amniotic growth factors such as IGF-1, and elevated cortisol levels resulting from hydrocortisone treatment for refractory hypotension. Additionally, incomplete knowledge of fetal ovine nutritional requirements may have contributed to the growth restriction observed [85]. Adverse outcomes of this model include fetal hydrops, evidence of white matter brain injury in supported lambs, and significantly reduced levels of IGF-1 [78,81].

#### 3.2.3. Neurologic Outcomes

Body and brain weights were similar between EVE animals and in utero controls. However, hydrops and edema were seen in animals on EVE, questioning whether the weight truly reflects somatic growth [85]. Evidence of gross white matter injury was reported in one of the seven animals, at the level of the basal ganglia (considered a watershed zone). No macroscopic histopathologic evidence of IVH was seen, even in younger fetal lambs and despite therapeutic anticoagulation [83].

Microscopic histologic analysis showed no increase in the density of Iba-1 microglia (marker for neuroinflammation) or olig2-positive (marker for oligodendrocyte lineage) cells in EVE animals without white matter injury [83]. Lambs on EVE received daily hydrocortisone to combat hypotension, despite the known interference with the growth hormone (GH) and insulin-like growth factor I (IGF-I) axes, the reduction in hippocampal neuronal density, and neurodegenerative-associated transcriptional changes [106,107]. These effects were not reported for lambs on EVE.

Neurodevelopmentally, all lambs on EVE performed fetal swallowing, breathing, and gross body movements at least once every six hours [83]. No neonatal post-EVE neurologic examination was reported.

### 3.3. Hospital Sant Joan de Déu, University of Barcelona (Spain)

#### 3.3.1. Model

The AWT system developed by Gratacós and colleagues is structurally similar to the EXTEND platform. It incorporates a pumpless arterio-venous circuit connected via umbilical cannulation, a low-resistance oxygenator, and a sterile fluid environment—semi-closed in design—where fetal lambs are fully immersed [101]. A detailed summary of this model’s specifications is provided in Table 1.

#### 3.3.2. Outcomes

In their latest publication, the team reported survival of up to 7 days for fetal lambs delivered at 110–115 days’ gestation, with an ultrasound-estimated weight of 1681 ± 77 g [101]. Their study included lambs supported on the AW system for various durations: 1–3 h, 4–24 h, and 48–168 h [101]. A significant learning curve was noted in transitioning lambs onto AW support, as reflected in the survival rates: 25% in the 1–3 h group, 70% in the 4–24 h group, and 80% in those maintained for 48–168 h [101]. The most frequent causes of mortality during cannulation or while on extracorporeal support included air embolism in the circuit, thrombosis, and technical malfunctions. As with other AW/AP systems, unfractionated heparin was administered for anticoagulation (see Table 1). In the group with the longest survival, physiologic circuit flow rates were achieved, along with normalization of lactate levels, pH balance, and heart rate.

#### 3.3.3. Neurologic Outcomes

No specific functional, structural, or biochemical neurologic outcomes were reported for this model. However, the study does highlight fetal hydrops as an adverse outcome in lamb fetuses maintained for 48–168 h on the system, a complication known to exacerbate hypoxia and cerebral hypoperfusion [101]. Similar to EVE, one concern is the use of hydrocortisone in this model, a treatment that may pose neurodevelopmental risks to the fetal brain [108,109].

### 3.4. Hospital for Sick Children, University of Toronto (Canada)

#### 3.4.1. Model

Haller and colleagues developed an AWT system to support fetal piglets, employing a pumpless arterio-venous circuit with umbilical cannulation, a low-resistance oxygenator, and a fully enclosed sterile fluid environment in which the piglets were completely submerged [102]. In a subsequent modification of the model, the team incorporated a centrifugal pump into the circuit to improve circulatory support [103].

#### 3.4.2. Outcomes

In their initial study, the researchers supported piglets delivered at 101 ± 6 days’ gestation, with an average weight of 651 ± 240 g. Of the 127 piglets included, 68 (53.5%) were successfully cannulated, and 12 of those (17.6%) were successfully transitioned onto the extracorporeal circuit [102]. These 12 piglets were maintained on support for an average of 28.5 ± 13.2 h, with the longest survival reaching 51 h (3077 min) [102]. After transitioning to AW support, umbilical vein (UV) flow rates dropped significantly compared to in utero measurements, and heart rates increased [102]. To address these hemodynamic changes, the group introduced a centrifugal pump in a follow-up study involving 13 piglets (born at 102 ± 4 days GA, average weight 616 ± 139 g) [103]. The pump led to enhanced UV flow and extended survival, with average support time increasing to 46.4 ± 46.8 h [103].

Common technical challenges included cannulation failure, accidental decannulation, UV vasospasm, thromboembolic complications, and, in the pumped configuration, instances of heart failure [102,103]. Notably, 4 of the 13 piglets (30.8%) supported with the pump died from heart failure [103]. Although the pump improved UV flow and helped decrease cardiac afterload, the suction it generated placed stress on the fetal heart by disrupting natural cardiac function and altering afterload balance factors, likely contributing to cardiac dysfunction and failure [110].

#### 3.4.3. Neurologic Outcomes

Studies in the piglet model have focused on cardiovascular performance and outcomes, with currently no reports on specific organ development, nor any functional, structural, or biochemical neurologic outcomes [102,103,104]. Similar to the model by the Barcelona team, fetal hydrops was reported.

### 3.5. University of Michigan (USA): Veno-Venous Premature ECLS

#### 3.5.1. Model

The original model developed by Mychaliska and colleagues in 2009 was an AWT system utilizing a pumpless arterio-venous circuit with umbilical vessel cannulation and full immersion of the fetus in fluid [77]. Although gas exchange was adequate, systemic hypotension and progressive cardiovascular collapse limited the survival of fetal lambs on this system to just four hours [77]. Even with the later addition of a pump to overcome circuit resistance, survival time did not improve significantly [68]. As a result, the team shifted their focus from an AWT model to a pump-assisted AP design. This revised approach employs a veno-venous circuit (VV-ECLS) with cannulation of the umbilical vein and the internal jugular vein [68]. Rather than full submersion, they simulate the intrauterine fluid environment by intubating the fetal lambs and filling and capping the endotracheal tubes with fluid. In the latest version of this model, a nitric oxide surface anticoagulation (NOSA) system, a non-thrombogenic circuit, was used, thus removing the need for systemic anticoagulation [111].

#### 3.5.2. Outcomes

Over time, the group reported average survival durations approaching two weeks on the circuit, although these experiments were conducted in more mature lambs (gestational age > 118 days EGA) [75]. At this developmental stage, a lamb’s lungs are roughly comparable to those of a 28-week human fetus [15]. In these models, the team demonstrated ongoing lung maturation during 7–10 days of support, with lung function post ventilation similar to age-matched controls [74,76]. It is important to note that the use of tracheal occlusion, achieved by sealing the fluid-filled endotracheal tubes, along with continuous corticosteroid administration, independently accelerates lung development [112,113,114], making it difficult to attribute pulmonary maturation solely to the AP support itself [59]. The addition of the NOSA system to the circuit resulted in successful seven-day support without thrombosis or significant bleeding, offering the hope of reducing or avoiding systemic anticoagulation all together [111].

#### 3.5.3. Neurologic Outcomes

No gross histologic findings of white matter injury or intracranial hemorrhage were reported in the 118-day-EGA lambs supported on VV-ECLS fir one week, regardless of whether systemic anticoagulation was used [72,111].

MRI analysis showed no difference in gyrification index (measure of cortical folding) between lambs on AP support and late-preterm (EGA 127) controls, suggesting physiological brain development on AP [72]. MRI enabled further white matter integrity assessment with fractional anisotropy and diffusion coefficient analysis in the frontal and parietal cortices, corpus callosum, and cerebral peduncles. Fractional anisotropy, a proxy for myelination, did not differ between animals supported on VV-ECLS and late-preterm controls [72].

Cerebrovascular function was assessed in the shorter 48 h study, reporting stable cerebral blood flow on the circuit comparable to controls, and intact CO2 reactivity as a marker for cerebral autoregulation [71].

No neurodevelopmental assessments were reported for the lambs on VV-ECLS or after delivery.

## 4. Discussion

Several preclinical AWT and AP systems have been shown to successfully reproduce key aspects of the intrauterine environment, enabling physiologic hemodynamical support and continued organ maturation. Heterogeneity in model design, gestational age at cannulation, the use of corticosteroids, and heparinization and nutritional strategies limit the generalization of outcomes across systems. Corticosteroid use independently affects inflammation, hemodynamics, and the risk of brain injury. Similarly, systemic anticoagulation to avoid thrombosis on extracorporeal circulation increases the risk of cerebral hemorrhage in EPIs [115]. Recognizing the risks of systemic anticoagulation and corticosteroids, research teams have made concerted efforts to avoid their use.

Overall, these models report no consistent hemorrhagic, ischemic, or inflammatory cerebral injury, with grossly preserved brain structure and continued cerebral maturation on the circuit. Nonetheless, important caveats remain regarding extrapolating these data to human EPIs. First, outcomes of different AWT models vary widely, with some groups reporting life-threatening adverse outcomes such as hydrops fetalis (Barcelona & Toronto) in some of their study animals and limited survival on the circuit (Table 1). In light of growing optimism about AWT, this reminds us that important development and research gaps surrounding the technology remain. Second, there are inherent differences between human and animal models, specifically the greater maturity of the ovine germinal matrix (protective against hemorrhagic injury) and the absence of long-term neurodevelopmental outcome data. While the absence of cerebral hemorrhage is definitely beneficial, we cannot assume that this will not manifest clinically in the context of the more immature and fragile human germinal matrix of EPIs. Furthermore, in contrast to altricial human infants, lambs are born precocial with greater neurological and behavioral maturity, rapidly demonstrating coordinated behaviors such as standing, locomotion, feeding readiness, and autonomic regulation, further marking differences in human and ovine neurodevelopmental maturity at birth. Third, the animal studies investigating current AWT models support otherwise healthy preterm lambs. In clinical reality, however, extremely preterm birth often occurs in the setting of underlying maternal–fetal pathology such as chorioamnionitis, pre-eclampsia, and placental insufficiency. These underlying pathologies can independently negatively impact neurodevelopment in EPIs. The preclinical data may therefore incompletely reflect the heterogeneous risk profiles and comorbidity burden among human EPIs.

Several ethical considerations have been raised in the literature regarding the clinical translation of AWT pertaining to balancing benefit and harm, parental autonomy and responsibility, the legal and moral status of the AWT subject, justice and access, cultural and societal perspectives, and research ethics and oversight [116,117]. While currently developed as an improved, more physiologic method of intensive care for EPIs, ethical speculation about futuristic use of AWT to achieve complete ectogenesis has captured the imagination of many philosophers and ethicists [118].

With first-in-human trials on the horizon, the absence of long-term neurodevelopmental outcome data in AWT and AP technology poses a significant challenge in counseling prospective parents considering participation in these experimental studies. Transparent, contextualized discussions about the available preclinical evidence, benchmarked against current outcomes achieved with standard NICU care, will be essential to ensure informed consent and to safeguard responsible, ethical clinical translation.

## Figures and Tables

**Table 1:** Overview of currently described preclinical AP and AW models.

	Artificial Placenta Model	Artificial Womb Model			
	Specifications				
**Group**	Ann Arbor, USA	Perth, Australia and Sendai, Japan	Philadelphia, USA	Barcelona, Spain	Toronto, Canada
**Model name**	VV preemie ECLS	Ex-Vivo uterine Environment (EVE)	EXTra-uterine Environment for Neonatal Development (EXTEND)	-	-
**Year of first publication of the current model (references using the current model)**	2009 [67,68,69,70,71,72,73,74,75,76,77]	2017 [78,79,80,81,82,83,84,85] ^§^	2017 [86,87,88,89,90,91,92,93,94,95,96,97,98,99,100]	2023 [101]	2021 [102,103,104]
**Species, GA at cannulation (range)**	Lambs, 130–135	Lambs, 95–115	Lambs, 95–117	Lambs, 110–115	Piglets, 91–106
**Circuit configuration, pump**	VV, roller pump	VA, pumpless	VA, pumpless	VA, pumpless	VA, centrifugal pump
**Cannulation, cannula size**	JV/UV (10–12 Fr)	UV/2 * UA (10/2 * 8 Fr)	UV/2 * UA (12/2 * 12 Fr)	UV/2 * UA (10–14 Fr)	UV/UA (2.1–3.3 mm)
**Fluid incubation (volume)**	No submersion; fluid-filled endotracheal tube	Sterile complete submersion (6 L)	Sterile complete submersion (2–4 L)	Semi-closed, complete submersion (10 L)	Sterile complete submersion (NS)
**Prophylactic use of antimicrobials**	Piperacillin-tazobactam, metronidazole, and fluconazole	Meropenem and fluconazole	No	Ceftazidime and meropenem; ultraviolet light sterilization	Piperacillin-tazobactam
**Anticoagulation drug (ACT goal)**	Heparin (200–250 s)	Heparin (180–220 s)	Heparin (150–180 s), no heparin [105]	Heparin (200–250 s)	Heparin (>300 s)
**Corticosteroids (Yes/No, type)**	Yes, methylprednisolone	Yes, hydrocortisone	No	Yes, hydrocortisone	Yes, hydrocortisone
**Other medications**	PGE1, erythropoietin, epinephrine (prn), norepinephrine (prn) and dopamine (prn), Diazepam (prn) and buprenorphine (prn).	Lipo-PGE1, Erythropoietin and Milrinone (first 24 h).	PGE1, Erythropoietin, insulin, buprenorphine (prn) and propofol (prn)	PGE1, pRBC	PGE1, papaverine, epinephrine (prn)
**Max. reported survival (reference), successful transition off AWT**	17 days [75], no	14 days [85], no	28 days [86], yes [86]	7 days [101], no	2 days [103], no
**Neurologic injury (IVH, WMI, hemorrhage, thrombosis)**	Absent [72]	WMI present in 1/7 animals (basal ganglia) [83]	Absent [86,96]	Fetal hydrops [101]	Fetal hydrops [102,103,104]
**Cerebral maturation**	MRI—comparable gyrification index and myelination to control [72]; stable cerebral blood flow and intact CO_2_ reactivity [71]	Comparable brain weight to control, though hydrops and edema present [85]	Myelin density and brain weight comparable to in utero controls [86,96]	Not reported	Not reported
**Histopathology**	No findings of WMI or hemorrhage [72]	No upregulation of Iba-1 or oligo2-positive cells [83]	Normal Iba-1 and cerebral arteriolar diameter [96]	Not reported	Not reported
**Transcriptomic analysis**	Not reported	Not reported	Comparable to age-matched late-preterm controls	Not reported	Not reported
**Neurodevelopmental outcomes**	Not reported	Performed fetal swallowing, breathing, and gross body movements	Consolidation of sleep–wake cycles on circuit [86]; no gross neurologic deficits; playful, food searching, and responsive to stimuli [96]	Not reported	Not reported

Abbreviations: GA = gestational age; VV = veno-venous; VA = veno-arterial; JV = jugular vein; UV = umbilical vein; UA = umbilical artery; ACT = activated clotting time; NS = not specified; prn = ‘pro re nata’ (when necessary); PGE1 = prostaglandin E1; pRBC = packed red blood cell; IVH = intraventricular hemorrhage; WMI = white matter injury. (^§^) = the groups most recently published study describes the use of a modified circuit (single oxygenator) and an adapted cannulation technique (UV intra-abdominal, 2 * UA extra-abdominal).

## Data Availability

No new data were created or analyzed in this study. Data sharing is not applicable to this article.

## Abbreviations

The following abbreviations are used in this manuscript:

WHO	World Health Organization
EPI	Extremely premature infant
EGA	Estimated gestational age
NICU	Neonatal intensive care
BPD	Bronchopulmonary dysplasia
VLBW	Very low birth weight
NEC	Necrotizing enterocolitis
ROP	Retinopathy of prematurity
IVH	Intraventricular hemorrhage
PVL	Periventricular leukomalacia
CP	Cerebral palsy
ADHD	Attention-deficit hyperactivity disorder
ASD	Autism spectrum disorder
AWT	Artificial womb technology
AP	Artificial placenta
GH	Growth hormone
IGF-1	Insulin-like growth factor
UV	Umbilical vein
VV-ECLS	Veno-venous circuit
NOSA	Nitric oxide surface anticoagulation
